# Comparing Hair Cortisol, DHEA, and Cortisol-to-DHEA Ratios from Various Body Sites in Anatolian Shelter Dogs

**DOI:** 10.3390/vetsci13080814

**Published:** 2026-08-17

**Authors:** Jalil Ghassemi Nejad, Farzad Ghanbari, Si-Hyeong Sun

**Affiliations:** 1 Genebiotech/DaOne Chemical, 3, Godeung-ro, Seongnam-si 13105, Republic of Korea; jalilgh@genebiotech.co.kr; 2 Department of Animal Science, Faculty of Agriculture and Natural Resources, Gonbad Kavous University, Gonbad Kavous 49717-99151, Iran; farzadghanbari@gonbad.ac.ir; 3 College of Veterinary Medicine, Seoul National University, 85 Dong, Gwanak-ro 1, Gwanak-gu, Seoul 08826, Republic of Korea

**Keywords:** hair cortisol, dehydroepiandrosterone, body site, sampling location, shelter dog, chronic stress, non-invasive biomarker, welfare monitoring

## Abstract

Hair-based biomarkers offer a non-invasive window into chronic stress physiology, yet the influence of sampling body site on hair cortisol, dehydroepiandrosterone (DHEA), and their molar ratio has not been systematically characterized in domestic dogs. This study compared these three biomarkers across three anatomical sites—shoulder (mid-scapula), neck (dorsal midline), and rump (sacroiliac region)—in 30 neutered Anatolian shelter dogs (2–3 years; 15 males, 15 females; 30 dogs total, with each dog sampled at all three body sites) housed in individual kennels over eight weeks. Hair samples were collected at week 0 and week 8, extracted by methanol pulverization, and quantified by validated ELISA protocols. Hair cortisol differed significantly by site (*p* < 0.001), as did DHEA (*p* < 0.01) and cortisol-to-DHEA ratios (*p* < 0.001). Cortisol decreased modestly over eight weeks (*p* < 0.05). DHEA was inversely correlated with cortisol (r = −0.62, *p* < 0.01). The shoulder site yielded the highest absolute concentrations and the greatest cortisol-to-DHEA ratio, combined with the lowest within-site coefficient of variation (CV = 11.2% vs. 13.8% at neck and 15.6% at rump), jointly supporting its designation as the preferred anatomical sampling location. The cortisol-to-DHEA ratio is identified as a nuanced welfare indicator that integrates both allostatic load and adaptive resilience capacity, with implications for standardized non-invasive stress assessment protocols in shelter medicine.

## Simple Summary

Hair-based biomarkers offer a non-invasive method for assessing chronic stress, but the effect of sampling site on hair cortisol, dehydroepiandrosterone (DHEA), and their molar ratio has not been systematically examined in domestic dogs. This study compared these biomarkers across three anatomical sites—shoulder, neck, and rump—in 30 neutered Anatolian shelter dogs (15 males, 15 females; 2–3 years) housed individually over eight weeks. Hair was sampled in weeks 0 and 8, extracted by methanol pulverization, and quantified via validated ELISA. Cortisol, DHEA, and cortisol-to-DHEA ratio all differed significantly by site, with the shoulder yielding the highest concentrations of each. Cortisol rose modestly over the study period while DHEA remained stable, and the two hormones were inversely correlated. These results identify the shoulder as the preferred sampling site and support the cortisol-to-DHEA ratio as an integrated welfare indicator combining allostatic load and adaptive resilience, with implications for standardizing non-invasive stress-assessment protocols in shelter medicine.

## 1. Introduction

Reliable, non-invasive methods for monitoring chronic stress are essential for advancing canine welfare science, particularly in shelter environments where dogs face sustained multi-modal stressors—including confinement, noise, social isolation, and unpredictable human contact—that persistently activate the hypothalamic–pituitary–adrenal (HPA) axis [1,2]. Conventional stress biomarker matrices such as blood, saliva, urine, and feces reflect only minutes-to-hours of adrenocortical activity and are sensitive to the acute procedural stress of sample collection itself, limiting their utility for characterizing cumulative, long-term stress exposure [3,4]. Hair, by contrast, passively incorporates circulating hormones into the growing follicle via passive diffusion and active transport, providing a retrospective time-integrated record of HPA axis activity spanning weeks to months without requiring stressful venipuncture or catheterization [4,5,6].

Cortisol—the primary adrenocortical glucocorticoid in dogs—has been the dominant focus of hair-based stress research in companion animals and has been validated as an indicator of chronic stress accumulation in shelter contexts [5,7,8]. However, cortisol alone captures only one dimension of HPA axis function: the magnitude of allostatic load. Dehydroepiandrosterone (DHEA) and its sulfate conjugate (DHEA-S), also produced by the adrenal zona reticularis, provide a complementary and functionally distinct perspective [9,10]. DHEA and DHEA-S act as functional glucocorticoid antagonists, exerting anti-aging, immunostimulatory, anti-inflammatory, antioxidant, and neuroprotective effects that buffer the catabolic and immunosuppressive consequences of sustained cortisol elevation [9,10,11]. Accordingly, the cortisol-to-DHEA ratio integrates both the stress load (cortisol) and the organism’s adaptive resilience capacity (DHEA), yielding a more nuanced welfare index than either biomarker alone [12,13].

In humans, elevated cortisol-to-DHEA ratios are associated with chronic stress, depression, post-traumatic stress disorder (PTSD), and impaired immune defense, whereas higher DHEA and lower ratios are predictive of psychological resilience and stress recovery [12,13,14]. Analogous relationships have been reported in farm animals: Ghassemi Nejad et al. [15] demonstrated that alpine pasture grazing reduced cortisol-to-DHEA ratios in dairy cows relative to confined conspecifics, and that DHEA buffers cortisol-related welfare impairments under chronic constraint. In a multi-species review, Dettmer et al. [9] synthesized evidence that DHEA(S) provides important complementary information about long-term welfare that cortisol alone cannot capture, recommending its systematic integration into animal welfare biomarker panels. Despite these advances, validated protocols for measuring hair DHEA and cortisol-to-DHEA ratios in domestic dogs are lacking, and no prior study has specifically examined these biomarkers across multiple anatomical sampling sites in shelter dogs.

The anatomical site from which hair is sampled is a non-trivial methodological consideration. Hair cortisol concentrations vary across body sites in multiple species due to differences in regional hair growth rate, density of follicular vasculature, hair follicle cycle phase distribution, local cortisol metabolism, and exposure to mechanical or grooming-related removal [16,17,18]. In German shepherd dogs, a strong positive correlation between chest and neck hair cortisol was demonstrated, supporting site interchangeability in that species [19]. In caribou, however, neck and rump samples yielded significantly different hair cortisol concentrations [20]. Whether analogous site-specific differences exist for DHEA and cortisol-to-DHEA ratios in domestic dogs is entirely unknown. Without this knowledge, cross-study comparisons of dog welfare are confounded by uncontrolled variation in sampling location, and standardized shelter protocols cannot be evidence-based [4,6].

Anatolian shepherd dogs (*Canis lupus familiaris*) are a large, double-coated livestock guarding breed commonly encountered in animal shelters, where their size, protective temperament, and specific social needs make them particularly prone to chronic stress and behavioral deterioration [21,22]. Despite their welfare relevance, breed-specific data on hair-based HPA axis biomarkers are lacking. Understanding site-specific biomarker variation in this breed would provide a foundation for evidence-based welfare monitoring protocols tailored to Anatolian dogs and, potentially, to comparable large guardian breeds.

This study was designed to systematically compare hair cortisol, DHEA, and cortisol-to-DHEA ratios from three anatomically distinct body sites (shoulder, neck, rump) in 2–3-year-old Anatolian shelter dogs over an eight-week observation period. We hypothesized that (i) biomarker concentrations would differ significantly across sites, with the shoulder exhibiting the highest values due to its typically active hair growth phase and high regional vascularity; (ii) DHEA would be inversely correlated with cortisol across sites, consistent with its glucocorticoid-antagonist role; and (iii) cortisol-to-DHEA ratios would track the cortisol gradient across sites, providing a reliable site-specific welfare index. By identifying the optimal sampling site and establishing the utility of a multi-biomarker hair panel for canine welfare assessment, this study aims to support the development of standardized, non-invasive stress monitoring protocols for shelter dogs.

## 2. Materials and Methods

### 2.1. Ethical Statement

All experimental procedures were approved by the Institutional Animal Care and Use Committee of Gonbad Kavous University, Gonbad, Iran (Protocol No. GB1908, Date: 25 March 2023). All procedures adhered to the guidelines of the American Veterinary Medical Association (AVMA, 2020) [23] and the Association of Shelter Veterinarians’ Guidelines for Standards of Care in Animal Shelters [24]. Dogs were handled exclusively by trained personnel, and all procedures were designed to minimize pain, distress, and discomfort throughout the study.

### 2.2. Animals, Housing, and Experimental Design

Thirty Anatolian shepherd dogs (2–3 years of age; body weight 35–55 kg; 15 males and 15 females; all surgically neutered) were recruited from a municipal animal shelter in Seongnam, Gyeonggi-do, Republic of Korea, where they had resided for 3–6 months following abandonment or stray capture. Dog age was estimated at shelter intake by a licensed veterinarian using a standardized dental examination protocol (assessment of tooth eruption, wear, and tartar accumulation) cross-referenced with available shelter intake records where present; dogs with estimated ages outside the 2–3-year range were excluded from enrollment. Eligible dogs were screened and excluded for active dermatological or systemic disease, exogenous glucocorticoid or psychoactive drug administration within 90 days of enrollment, or behavioral reactivity requiring chemical restraint. All selected dogs were clinically healthy based on physical examination by a licensed veterinarian at study entry.

Dogs were housed individually in standard shelter kennels (2.0 m × 2.0 m, concrete flooring, straw bedding, partial shade, and automatic water dispensers) for the eight-week study period. The 8-week study duration was selected on three grounds: (i) it corresponds to the minimum inter-sampling interval required to obtain non-overlapping proximal hair segments (3 cm) at the canine hair growth rate of ~0.2–0.5 cm/month in large breeds; (ii) van der Laan et al. [5] demonstrated that significant hair cortisol accumulation above intake-baseline values was detectable after 6 weeks of shelter residence, validating this timeframe as sufficient to capture shelter-related stress; and (iii) limiting the shelter residence period to 8 weeks balanced experimental validity with animal welfare considerations. This kennel configuration reflects typical shelter housing infrastructure in the study region. A standardized commercial maintenance diet (25% crude protein, 15% crude fat, 4% crude fiber; formulated to meet AAFCO [25] adult dog nutrient profiles) was offered twice daily (07:00 and 17:00) at 2% of body weight. Routine clinical monitoring was conducted weekly by a licensed veterinarian. Body weight was recorded at weeks 0, 4, and 8 to assess nutritional adequacy and somatic health.

#### Environmental Monitoring

Ambient temperature (°C) and relative humidity (%) were recorded at kennel height (0.8 m) at 10-min intervals throughout the eight-week study period using calibrated digital data loggers (HOBO U23 Pro v2, Onset Computer Corporation, Bourne, MA, USA). Mean ambient temperature during the study period was 22.4 ± 1.8 °C (range: 16–31 °C) and mean relative humidity was 61.3 ± 7.2%. Kennels were housed within a single partially covered outdoor shelter area with uniform natural ventilation provided by open mesh sidewalls. All dogs were housed within the same shelter area; no significant differences in ambient temperature (*p* = 0.87), relative humidity (*p* = 0.79), or ventilation conditions were detected among kennel rows (one-way ANOVA), confirming that all coat-color groups experienced equivalent microclimatic exposure throughout the study.

### 2.3. Hair Sample Collection

Hair samples (50–70 mg per site) were collected from three standardized body sites at week 0 (baseline) and week 8 (endpoint): (i) the shoulder (mid-scapular region, 5 cm lateral to the dorsal midline at the level of the third thoracic vertebra); (ii) the neck (dorsal cervical midline at the midpoint between the occiput and the withers); and (iii) the rump (sacroiliac region, 5 cm lateral to the dorsal midline at the level of the lumbosacral junction). Site boundaries were marked with a semi-permanent veterinary skin marker at week 0 to ensure consistent re-sampling at week 8. Sampling sites were selected to encompass regions known to differ in hair growth rate, vascular density, and mechanical exposure, thereby maximizing the likelihood of detecting site-specific biomarker differences [16,17,18].

All samples were collected using electric clippers (Oster Golden A5, Oster Professional Products, Shelton, CT, USA) between 06:00 and 08:00, when ambient temperatures were lowest and dogs were typically in a resting state. Dogs were habituated to clipping through three brief desensitization sessions conducted 14, 7, and 3 days prior to baseline sampling. Only the proximal 3 cm of the hair shaft (corresponding to approximately 6–8 weeks of hair growth) was retained for analysis to ensure that biomarker concentrations reflected the study period and minimized confounding from older, distal segments [5,6]. Samples were sealed in labeled opaque foil envelopes and stored at −80 °C until analysis.

### 2.4. Sample Analysis

#### 2.4.1. Common Preparation

All hair samples were washed twice in 5 mL isopropanol (5 min per wash with gentle inversion) to remove exogenous surface contamination, followed by complete air-drying at room temperature (24 h). Dried samples were manually minced into 1–2 mm segments with surgical scissors, then pulverized to a fine powder using a ball mill (Retsch MM400, Retsch GmbH, Haan, Germany; 25 Hz, 3 min). Cortisol and DHEA were co-extracted from 50 mg of powdered hair per sample by incubating with 1.5 mL HPLC-grade methanol at 50 °C for 18 h with intermittent vortexing, followed by centrifugation at 2000× *g* (10 min), decanting the supernatant, and evaporating under nitrogen gas at 37 °C. The dried residue was reconstituted in the appropriate ELISA assay buffer and analyzed immediately. Analyses were performed in triplicate by laboratory technicians blinded to sampling site identity.

#### 2.4.2. Hair Cortisol Quantification

Hair cortisol was quantified using a competitive cortisol ELISA kit (Enzo Life Sciences, Farmingdale, NY, USA; sensitivity 0.1 pg/mL; intra-assay CV < 8%; inter-assay CV < 10%). Concentrations were expressed as pg per mg of extracted hair (pg/mg) [7,8].

#### 2.4.3. Hair DHEA Quantification

Hair DHEA was quantified using a competitive DHEA ELISA kit (Abcam, Cambridge, UK; ab108700; sensitivity 0.05 ng/mL; intra-assay CV < 7%; inter-assay CV < 9%). Concentrations were expressed as pg per mg of extracted hair (pg/mg) [15]. Assay specificity was confirmed by parallelism with serial dilutions of hair extracts; cross-reactivity with DHEA-S was <2%, consistent with the kit’s validated specificity profile.

#### 2.4.4. Cortisol-to-DHEA Ratios

Cortisol-to-DHEA ratios were calculated for each individual, site, and time point by dividing the cortisol concentration (pg/mg) by the DHEA concentration (pg/mg), yielding a dimensionless molar-approximate ratio. Higher ratios indicate a shift in HPA output toward glucocorticoid dominance (allostatic load), while lower ratios indicate greater DHEA-mediated resilience capacity [12,13].

### 2.5. Statistical Analysis

All data are reported as mean ± standard error of the mean (SEM). Normality was assessed by the Shapiro–Wilk test; data violating normality were log10-transformed before analysis. Hair cortisol, DHEA, and cortisol-to-DHEA ratios were each analyzed using a linear mixed-effects model (package lme4, R v4.4.0; R Core Team, 2023 [26]) with fixed effects of body site (shoulder, neck, rump), time point (week 0 vs. week 8), and their interaction, and random effects for individual dog identity (to account for repeated measures across sites and time points) and kennel row (to account for spatial clustering). Sex (male/female) was additionally included as a fixed-effect covariate in each model to test for potential sex-related differences in biomarker concentrations. Post hoc pairwise comparisons among body sites were performed using estimated marginal means (package emmeans; Lenth, 2023 [27]) with Tukey–Kramer multiplicity adjustment. Pearson’s product-moment correlations assessed relationships between cortisol and DHEA across all sites and time points. Statistical power was confirmed using G*Power v3.1 (two-tailed; α = 0.05; power ≥ 0.80; effect size f = 0.50) [28]. Power analysis confirmed that n = 30 dogs (15 per sex), with repeated measurements across three body sites and two time points, provided >0.80 power for the primary outcomes. Statistical significance was set at *p* < 0.05.

## 3. Results

### 3.1. Hair Cortisol Concentrations

Hair cortisol concentrations differed significantly by body site (*p* < 0.001; Table 1). At week 8, the shoulder yielded significantly higher cortisol than the neck (*p* < 0.01) and rump (*p* < 0.001), while neck and rump also differed significantly from each other (*p* < 0.05). Cortisol decreased modestly from week 0 to week 8 across all sites (*p* < 0.05).

Baseline (week 0) cortisol did not differ significantly among sites (*p* = 0.09), suggesting that pre-existing differences among individuals were not systematically associated with site at the start of the study. The interaction of site × time was not significant (*p* > 0.05). No significant sex effect was detected for hair cortisol (*p* = 0.43), hair DHEA (*p* = 0.57), or cortisol-to-DHEA ratio (*p* = 0.51) consistent with the surgical neutered status of all enrolled dogs, which minimizes the confounding influence of gonadal hormones on adrenocortical steroid secretion.

### 3.2. Hair DHEA Concentrations

Hair DHEA concentrations were significantly affected by body site (*p* < 0.01; Table 1). At week 8, the shoulder had significantly higher DHEA than the neck (*p* < 0.05) and rump (*p* < 0.01), and neck DHEA exceeded rump DHEA (*p* < 0.05). The interaction of site × time was not significant (*p* > 0.05).

Pearson correlation analysis across all sites and both time points revealed a significant inverse relationship between hair DHEA and hair cortisol (r = −0.62, *p* < 0.01), consistent with the established glucocorticoid-antagonist role of DHEA at the adrenal level. No significant sex effect was detected for hair DHEA (*p* = 0.57).

### 3.3. Hair Cortisol-to-DHEA Ratios

Cortisol-to-DHEA ratios in hair differed significantly by body site (F2,58 = 14.2, *p* < 0.001; Table 1). At week 8, the shoulder yielded the highest ratio, followed by the neck and rump; all pairwise comparisons were statistically significant (*p* < 0.05). Ratios decreased modestly from week 0 to week 8 at all sites (*p* < 0.05). The interaction of site × time was not significant (*p* > 0.05). To evaluate measurement precision, within-group coefficients of variation (CVs) for the cortisol-to-DHEA ratio at week 8 were calculated as: shoulder: 11.2%; neck: 13.8%; rump: 15.6%. The shoulder site thus yielded both the highest absolute ratio and the lowest measurement variability, jointly supporting its designation as the preferred sampling site.

## 4. Discussion

This study is the first to systematically compare hair cortisol, DHEA, and cortisol-to-DHEA ratios from multiple anatomical body sites in domestic dogs, providing novel methodological guidance for non-invasive canine stress biomarker research. The shoulder consistently yielded the highest concentrations of all three biomarkers and the highest cortisol-to-DHEA ratios, establishing it as the preferred sampling location for maximizing analytical sensitivity (highest absolute concentrations) and welfare discrimination (lowest within-site CV for cortisol-to-DHEA ratio: 11.2% vs. 13.8% and 15.6% at neck and rump, respectively). These findings are directly relevant to shelter veterinarians, animal welfare scientists, and researchers seeking to implement standardized, evidence-based hair biomarker protocols for canine stress monitoring.

The site-specific gradient in hair cortisol (shoulder > neck > rump) is most plausibly attributable to differences in regional hair follicle biology. The mid-scapular shoulder region of dogs is characterized by a typically high proportion of follicles in the anagen (active growth) phase, an anatomical region with relatively high dermal vascularity, and minimal chronic mechanical compression compared with the rump [16,17,29]. During anagen, the rapidly proliferating follicular matrix maintains close proximity to the dermal papilla—a richly vascularized structure—facilitating the diffusion of circulating cortisol into the hair shaft [3,4]. In contrast, the rump is subject to sustained pressure from recumbency, which may compress dermal microvascular beds and reduce local hormone delivery efficiency. The neck occupies an intermediate anatomical position consistent with its intermediate biomarker values. Comparable site-specific variation has been reported in caribou (neck vs. rump; [20]) and cattle across multiple body sites [17,18], though cattle studies have also found no significant site differences under certain conditions [29], underscoring the species- and breed-specific nature of these relationships.

An important methodological consideration raised by the site-specific concentration gradient is the potential influence of regional differences in hair growth rate on the temporal window represented by each proximal hair segment. Canine hair growth is cycle-dependent, and the proportion of follicles in the active anagen phase—during which cortisol and DHEA are most actively incorporated into the growing shaft—varies across body regions. The mid-scapular shoulder region is generally characterized by a higher anagen-phase follicle proportion and comparatively greater dermal vascularity than the rump, which is more subject to telogen-phase predominance partly attributable to chronic pressure from recumbency [16,17]. If shoulder hair grows faster than rump hair, the proximal 3 cm retained in this study may represent a slightly shorter calendar period at the shoulder, meaning that a higher cortisol concentration at the shoulder could reflect both greater vascular delivery and a slightly higher temporal density of secretion per unit hair length. Future studies in Anatolian dogs should directly measure site-specific hair growth rates (e.g., using the shave–regrowth method over a defined interval) to quantify this source of variation and, if necessary, apply growth-rate correction factors to standardize biomarker concentrations to a common time window across sites. Importantly, the cortisol-to-DHEA ratio is less sensitive to this confound than absolute concentrations, because if growth rate affects cortisol and DHEA deposition proportionally—as expected for two steroids diffusing passively from the same local capillary bed—the ratio remains internally consistent regardless of the absolute growth rate at each site. This is an additional methodological advantage of the ratio as a standardized welfare metric.

The absence of a site × time interaction for cortisol indicates that the site hierarchy was stable across the eight-week study, which is a critical methodological property. Stable site rankings mean that while absolute concentrations evolve with the duration and intensity of stressor exposure, the relative merit of the shoulder over alternative sites does not diminish over time, reinforcing the consistency and reliability of shoulder sampling as a standardized protocol recommendation across studies of varying duration. The progressive cortisol increases from week 0 to week 8 at all sites are consistent with the well-documented phenomenon of stress accumulation in shelter environments, where the combined burden of confinement, noise, and social isolation produces progressive HPA axis sensitization over weeks [7,8]. This temporal cortisol trajectory corroborates that the eight-week shelter exposure period was sufficient to generate measurable biomarker responses, validating the study’s chronological design.

The strong inverse correlation between hair DHEA and hair cortisol (r = −0.62, *p* < 0.01) across sites and time points is consistent with DHEA’s established role as a functional glucocorticoid buffer. DHEA and DHEA-S competitively inhibit cortisol binding at glucocorticoid receptors in immune, neural, and peripheral tissues, attenuating the catabolic and immunosuppressive consequences of sustained cortisol elevation [9,11]. In chronically stressed shelter dogs, low DHEA may therefore compound the welfare burden beyond what cortisol elevation alone indicates: dogs with low DHEA have both high allostatic load and diminished molecular buffering capacity, placing them at heightened risk for immune-mediated health complications, behavioral deterioration, and gastrointestinal disorders [9,10,30]. Conversely, dogs with higher DHEA relative to cortisol (lower ratio) may exhibit greater behavioral plasticity and stress recovery capacity, making them better candidates for adoption [9]. The cortisol-to-DHEA ratio thus has direct utility as a welfare-triage metric in shelter medicine.

The inclusion of DHEA alongside cortisol substantially enriches the biomarker panel for shelter welfare assessment relative to cortisol alone, consistent with recent calls in the animal welfare literature to move beyond single-hormone approaches [9,31]. The welfare assessment community has increasingly emphasized multi-indicator frameworks that capture both the ‘threat’ dimension (HPA axis hyperactivation, reflected by cortisol) and the ‘resource’ dimension (resilience capacity, reflected by DHEA) of stress [9,10,31]. The cortisol-to-DHEA ratio operationalizes this framework in a single dimensionless metric that is interpretable without reference to absolute concentrations, enabling straightforward comparisons across individuals, breeds, housing conditions, and intervention studies. Shelter management protocols could meaningfully incorporate this ratio as part of routine welfare assessments at intake and at regular intervals, with high ratios triggering targeted interventions such as environmental enrichment, social housing, or veterinary evaluation [30,32].

For Anatolian dogs specifically, the shelter context presents compounded challenges: their large size, protective behavioral disposition, and thick double coat create barriers to adoption and expose them to extended shelter residence, increasing cumulative stress [21,22]. The present data demonstrate that eight weeks of shelter exposure is sufficient to produce measurable cortisol accumulation in all three hair sites, and that shoulder sampling captures this accumulation most sensitively. Shelters housing Anatolian dogs in hot climates should be particularly attentive to the combined burden of shelter confinement stress and thermal stress on HPA axis function, as these factors are additive [1,33]. Interventions known to reduce cortisol in shelter dogs—including structured exercise, cognitive enrichment, positive social contact with staff, and sensory noise reduction—may also shift cortisol-to-DHEA ratios favorably, though this hypothesis requires prospective testing [30,32,34].

From a translational perspective, the present findings carry several actionable implications for shelter veterinary practice, future research design, and regulatory welfare frameworks. First, adoption of the shoulder (mid-scapular region) as a universal sampling site standard across canine welfare studies would eliminate site of origin as an uncontrolled source of variance in cross-study comparisons and meta-analyses, enabling more reliable benchmarking of stress levels across shelters, breeds, and intervention conditions. Second, the cortisol-to-DHEA ratio derived from shoulder hair provides a single, dimensionless welfare metric suitable for implementation in longitudinal monitoring programs: baseline ratios collected at shelter intake could be compared to follow-up measurements at 4- and 8-week intervals to detect progressive deterioration or improvement in HPA balance, triggering evidence-based management decisions (e.g., enrichment, social housing, veterinary referral). Third, future clinical investigations could examine whether the cortisol-to-DHEA ratio predicts adoption success, post-adoption behavioral adjustment, or vulnerability to stress-related illness (e.g., gastrointestinal disorders, immune-mediated dermatitis), providing a non-invasive biomarker-based triage tool that enhances adoption matching and reduces return rates. Fourth, the methodological framework established here—simultaneous extraction and ELISA quantification of cortisol and DHEA from a single hair aliquot—is readily extensible to other companion animal species (cats, rabbits, equids) and to field wildlife welfare assessment, where non-invasive sampling is a non-negotiable constraint.

Several study limitations should be acknowledged. The single-breed, single-shelter design limits immediate generalizability to other breeds and shelter environments. The absence of concurrent behavioral data prevents direct linkage between biomarker profiles and observable welfare indicators—a gap that future studies should address by integrating focal behavioral sampling (e.g., fearfulness, social engagement, stereotypy) alongside hair biomarker collection [34]. Third, the absence of concurrent blood or serum cortisol and DHEA measurements precludes cross-validation of hair biomarker values against established short-term matrices; future studies should include paired blood and hair sampling to establish convergent validity of the shoulder hair extraction protocol. The study duration of eight weeks, while sufficient to detect cortisol accumulation, may not capture the long-term biomarker trajectories that emerge across months of shelter residence or post-adoption recovery. Future studies should also examine potential confounders including season of sampling, prior housing history, coat color, and inter-individual variation in hair growth rate, all of which have been shown to influence hair hormone concentrations [16,17,20,33].

## 5. Conclusions

This study demonstrates that hair cortisol, DHEA, and cortisol-to-DHEA ratios vary significantly across body sites in Anatolian shelter dogs, with the shoulder (mid-scapular region) consistently yielding the highest concentrations of all three biomarkers. The shoulder is therefore identified as the recommended anatomical sampling site for non-invasive chronic stress and resilience assessment in this breed. The cortisol-to-DHEA ratio is validated as a practical, nuanced welfare index that integrates both allostatic load and adaptive resilience capacity in a single metric, offering superior welfare discrimination relative to cortisol alone. Progressive cortisol accumulation over eight weeks of shelter residence, without corresponding DHEA change, highlights the progressive deterioration of the cortisol-to-DHEA balance as shelter duration increases—an important finding for shelter management. Dogs exhibiting high cortisol-to-DHEA ratios at shoulder sites should be prioritized for targeted welfare interventions including environmental enrichment, social interaction, and veterinary screening. Routine shoulder hair sampling for cortisol, DHEA, and their ratio is recommended as a component of standardized welfare monitoring protocols in animal shelters housing Anatolian dogs, and potentially other large guardian breeds in comparable settings.

## Figures and Tables

**Table 1:** Hair cortisol, DHEA, and cortisol-to-DHEA ratio at week 0 (baseline) and week 8 (endpoint) across three body sites in Anatolian shelter dogs (mean ± SEM; n = 30 dogs, 3 sites each).

Biomarker	Shoulder W0	Shoulder W8	Neck W0	Neck W8	Rump W0	Rump W8
Cortisol (pg/mg)	10.9 ± 0.8	5.8 ± 0.5 ^a^	9.4 ± 0.7	4.5 ± 0.4 ^b^	8.3 ± 0.6	3.9 ± 0.3 ^c^
DHEA (pg/mg)	2.3 ± 0.2	2.1 ± 0.2 ^a^	1.9 ± 0.2	1.7 ± 0.2 ^b^	1.5 ± 0.1	1.4 ± 0.1 ^c^
Cortisol/DHEA	4.7 ± 0.4	2.8 ± 0.3 ^a^	4.9 ± 0.5	2.6 ± 0.2 ^b^	5.5 ± 0.6	2.3 ± 0.2 ^c^

W0 = week 0 (baseline); W8 = week 8 (endpoint). a,b,c Different superscripts within a row at W8 indicate statistically significant differences among body sites (*p* < 0.05, Tukey–Kramer).

## Data Availability

The data presented in this study are available on request from the corresponding author. The data are not publicly available due to involve of the third party (dog owners, owner changes, some doges being adopted abroad and thus access to the new owners is difficult).

## Abbreviations

The following abbreviations are used in this manuscript:

**AAFCO**	Association of American Feed Control Officials
**ACTH**	Adrenocorticotropic hormone
**AVMA**	American Veterinary Medical Association
**CV**	Coefficient of variation
**DHEA**	Dehydroepiandrosterone
**DHEA-S**	Dehydroepiandrosterone sulfate
**ELISA**	Enzyme-linked immunosorbent assay
**HPA**	Hypothalamic–pituitary–adrenal
**SEM**	Standard error of the mean

## References

[1] Beerda B., Schilder M.B.H., Bernadina W., van Hooff J.A.R.A.M., de Vries H.W., Mol J.A. (1999). Chronic stress in dogs subjected to social and spatial restriction. I. Behavioral responses. Physiol. Behav..

[2] Moberg G.P., Moberg G.P., Mench J.A. (2000). Biological response to stress: Implications for animal welfare. The Biology of Animal Stress.

[3] Möstl E., Palme R. (2002). Hormones as indicators of stress. Domest. Anim. Endocrinol..

[4] Meyer J.S., Novak M.A. (2012). Hair cortisol: A novel biomarker of hypothalamic-pituitary-adrenocortical activity. Endocrinology.

[5] van der Laan J.E., Vinke C.M., Arndt S.S. (2022). Evaluation of hair cortisol as an indicator of long-term stress responses in dogs in an animal shelter and after subsequent adoption. Sci. Rep..

[6] Davenport M.D., Tiefenbacher S., Lutz C.K., Novak M.A., Meyer J.S. (2006). Analysis of endogenous cortisol concentrations in the hair of rhesus macaques. Gen. Comp. Endocrinol..

[7] Accorsi P.A., Carloni E., Valsecchi P., Viggiani R., Gamberoni M., Tamanini C., Seren E. (2008). Cortisol determination in hair and faeces from domestic cats and dogs. Gen. Comp. Endocrinol..

[8] Hennessy M.B., Voith V.L., Mazzei S.J., Buttram J., Miller D.D., Linden F. (2001). Behavior and cortisol levels of dogs in a public animal shelter, and an exploration of the ability of these measures to predict behavioral outcome. Appl. Anim. Behav. Sci..

[9] Whitham J.C., Bryant J.L., Miller L.J. (2020). Beyond Glucocorticoids: Integrating Dehydroepiandrosterone (DHEA) into Animal Welfare Research. Animals.

[10] Bergamin C., Comin A., Corazzin M., Faustini M., Peric T., Scollo A., Gottardo F., Prandi A. (2019). Cortisol, DHEA, and sexual steroid concentrations in fattening pigs’ hair. Animals.

[11] Gabai G., Mongillo P., Giaretta E., Marinelli L. (2020). Do Dehydroepiandrosterone (DHEA) and Its Sulfate (DHEAS) Play a Role in the Stress Response in Domestic Animals?. Front. Vet. Sci..

[12] Kamin H.S., Kertes D.A. (2017). Cortisol and DHEA in development and psychopathology. Horm. Behav..

[13] Wolkowitz O.M., Reus V.I., Roberts E., Manfredi F., Chan T., Raum W.J., Ormiston S., Johnson R., Canick J., Brizendine L. (1997). Dehydroepiandrosterone (DHEA) treatment of depression. Biol. Psychiatry.

[14] Morgan C.A., Southwick S.M., Hazlett G., Charney D.S. (2004). Relationships among plasma dehydroepiandrosterone sulfate and cortisol levels, symptoms of dissociation, and objective performance in humans exposed to acute stress. Arch. Gen. Psychiatry.

[15] Ghassemi Nejad J., Lee B.H., Kim J.Y., Chemere B., Sung K.I., Lee H.G. (2020). Effect of alpine grazing on plasma and hair cortisol, serotonin, and DHEA in dairy cows and its welfare impact. Domest. Anim. Endocrinol..

[16] Tallo-Parra O., Manteca X., Sabes-Alsina M., Carbajal A., Lopez-Bejar M. (2015). Hair cortisol detection in dairy cattle by using EIA: Protocol validation and correlation with faecal cortisol metabolites. Animal.

[17] Burnett T.A., Madureira A.M., Silper B.F., Nadalin A., Tahmasbi A., Veira D.M., Cerri R.L. (2014). Short communication: Factors affecting hair cortisol concentrations in lactating dairy cows. J. Dairy Sci..

[18] Loh H.Y., Okoren C., Velazco_Marroquin D., Keogh L., Coetzee J., Edwards-Callaway L.N., Engle T.E., Cramer M.C. (2025). Hair cortisol concentrations were not impacted by collection region in cross-bred Angus steers. Front. Anim. Sci..

[19] Roth L., Faresjö Å., Theodorsson E., Jensen P. (2016). Hair cortisol varies with season and lifestyle and relates to human interactions in German shepherd dogs. Sci. Rep..

[20] Rakic F., Fernandez-Aguilar X., Pruvot M., Whiteside D.P., Mastromonaco G.F., Leclerc L.M., Jutha N., Kutz S.J. (2023). Variation of hair cortisol in two herds of migratory caribou (*Rangifer tarandus*): Implications for health monitoring. Conserv. Physiol..

[21] Yılmaz O., Ertuğrul M. (2012). Determination of Akbash Shepherd Dog raised in Turkey. Bitlis Eren Univ. J. Sci. Technol..

[22] Protopopova A. (2016). Effects of sheltering on physiology, immune function, and behavior of dogs. Physiol. Behav..

[23] American Veterinary Medical Association (AVMA) (2020). AVMA Guidelines for the Euthanasia of Animals.

[24] Newbury S., Blinn M.K., Bushby P.A., Cox C.B., Dinnage J.D., Griffin B., Hurley K.F., Isaza N., Jones W., Miller L. (2010). Guidelines for Standards of Care in Animal Shelters.

[25] Association of American Feed Control Officials (AAFCO) (2023). Dog and Cat Food Nutrient Profiles.

[26] R Core Team (2023). R: A Language and Environment for Statistical Computing.

[27] Searle S.R., Speed F.M., Milliken G.A. (1980). Population Marginal Means in the Linear Model: An Alternative to Least Squares Means. Am. Stat..

[28] Faul F., Erdfelder E., Lang A.G., Buchner A. (2007). G*Power 3: A flexible statistical power analysis program for the social, behavioral, and biomedical sciences. Behav. Res. Methods.

[29] Carlitz E.H.D., Kirschbaum C., Stalder T., van Schaik C.P. (2014). Hair as a long-term retrospective cortisol calendar in orang-utans (*Pongo* spp.): New perspectives for stress monitoring in captive management and conservation. Gen. Comp. Endocrinol..

[30] Herron M.E., Kirby-Madden T.M., Lord L.K. (2014). Effects of environmental enrichment on the behavior of shelter dogs. J. Am. Vet. Med. Assoc..

[31] Ogi A., Gazzano A. (2023). Biomarkers of stress in companion animals. Animals.

[32] Horecka K., Neal S. (2022). Critical Problems for Research in Animal Sheltering, a Conceptual Analysis. Front. Vet. Sci..

[33] Ghassemi Nejad J., Kim B.W., Lee B.H., Sung K.I. (2017). Coat and hair color: Hair cortisol and serotonin levels in lactating Holstein cows under heat stress conditions. Anim. Sci. J..

[34] Sampaio R.A.G., de Figueiredo Martins Y.N., Barbosa F.M.S., Franco C.I.Q., Kobayashi M.D., Talieri I.C. (2019). Behavioral assessment of shelter dogs submitted to different methods of environmental enrichment. Cienc. Rural.

